# High tumour contamination of leukaphereses in patients with small cell carcinoma of the lung: a comparison of immunocytochemistry and RT-PCR

**DOI:** 10.1054/bjoc.2001.2177

**Published:** 2001-11

**Authors:** L Perey, J Benhattar, R Peters, P Jaunin, S Leyvraz

**Affiliations:** Centre Pluridisciplinaire d'Oncologie and the Institut de Pathologie, Centre Hospitalier Universitaire Vaudois, Lausanne, Switzerland

**Keywords:** tumour cell contamination, leucaphereses, SCLC, RT-PCR, immunocytochemistry staining

## Abstract

In small-cell lung carcinoma (SCLC) tumour cell contamination of leukaphereses is unknown. The present study was performed to define appropriate markers for reverse transcriptase polymerase chain reaction (RT-PCR), then to assess the contamination rate of leukaphereses and corresponding bone marrow samples. Immunocytochemistry (ICC) and RT-PCR methods were also compared. Among the 33 patients included, analyses were performed in 16 who had multiple leukaphereses and 17 who had only bone marrow. Leukapheresis products and bone marrow were analysed by ICC using several specific monoclonal antibodies against neural-cell adhesion molecule (N-CAM), epithelial glycoprotein (EGP-40) and cytokeratins (CK). Samples were also analyzed by RT-PCR for expression for N-CAM, synaptophysin, neuron-specific enolase, chromogranin, cytokeratin-18/-19, CEA, EGP-40, apomucin type 1 (MUC-1) and human endothelial cell-specific molecule (ESM-1). Using ICC staining, contaminating tumour cells were detected in 34% of leukaphereses (27% in patients with limited disease and 43% in those with extensive disease). N-CAM was the most reliable marker for detection of contamination. For RT-PCR, CK-19 and CEA were the only appropriate markers. Positive signal rate in leukaphereses increased to 78% (89% for patients with limited disease and 67% for extensive disease). In bone marrow, both techniques were in agreement whereas in leukaphereses, RT-PCR was better than ICC. A high rate of tumour cell contamination was demonstrated not only in bone marrow but also in leukaphereses from SCLC patients. The most appropriate technique was RT-PCR mainly in patients with limited disease. © 2001 Cancer Research Campaign http://www.bjcancer.com


*British Journal of Cancer* (2001) **85**(11), 1713–1721
© 2001 Cancer Research Campaign
doi: 10.1054/ bjoc.2001.2177, available online at http://www.idealibrary.com on IDEAL

http://www.bjcancer.com

### High tumour contamination of leukaphereses in patients with small cell carcinoma of the lung: a comparison of immunocytochemistry and RT-PCR

**L Perey, J Benhattar, R Peters, P Jaunin and S Leyvraz**

Centre Pluridisciplinaire d'Oncologie and the Institut de Pathologie, Centre Hospitalier Universitaire Vaudois, Lausanne, Switzerland

**Summary** In small-cell lung carcinoma (SCLC) tumour cell contamination of leukaphereses is unknown. The present study was performed to define appropriate markers for reverse transcriptase polymerase chain reaction (RT-PCR), then to assess the contamination rate of leukaphereses and corresponding bone marrow samples. Immunocytochemistry (ICC) and RT-PCR methods were also compared. Among the 33 patients included, analyses were performed in 16 who had multiple leukaphereses and 17 who had only bone marrow. Leukapheresis products and bone marrow were analysed by ICC using several specific monoclonal antibodies against neural-cell adhesion molecule (N-CAM), epithelial glycoprotein (EGP-40) and cytokeratins (CK). Samples were also analyzed by RT-PCR for expression for N-CAM, synaptophysin, neuron-specific enolase, chromogranin, cytokeratin-18/-19, CEA, EGP-40, apomucin type 1 (MUC-1) and human endothelial cell-specific molecule (ESM-1). Using ICC staining, contaminating tumour cells were detected in 34% of leukaphereses (27% in patients with limited disease and 43% in those with extensive disease). N-CAM was the most reliable marker for detection of contamination. For RT-PCR, CK-19 and CEA were the only appropriate markers. Positive signal rate in leukaphereses increased to 78% (89% for patients with limited disease and 67% for extensive disease). In bone marrow, both techniques were in agreement whereas in leukaphereses, RT-PCR was better than ICC. A high rate of tumour cell contamination was demonstrated not only in bone marrow but also in leukaphereses from SCLC patients. The most appropriate technique was RT-PCR mainly in patients with limited disease. © 2001 Cancer Research Campaign http://www.bjcancer.com

**Keywords**: tumour cell contamination; leucaphereses; SCLC; RT-PCR; immunocytochemistry staining

Small-cell lung carcinoma (SCLC) accounts for 20–25% of all bronchogenic carcinomas, has a short doubling time and a strong metastatic potential (Weiss et al, 1970; Muggia et al, 1974). Among the various possible approaches to improving the outcome of SCLC patients, intensification of chemotherapy is a promising option. Early clinical results suggested some impact of the dose of the cytostatic agent on the outcome (Ettinger et al, 1978; Weiss, 1978). In recent years, randomized trials were performed to study early intensification regimens with increases in dose intensity varying between 7% and 33% (Arriagada et al, 1993; Woll et al, 1995; Steward et al, 1998; Thatcher et al, 2000). These studies did reveal encouraging improvements in 2-year survival. High-dose chemotherapy with autologous stem cell support administered upfront resulted in impressive rates of complete remission (56–67%) among patients with limited disease (Farha et al, 1983; Souhami et al, 1985; Pettengell et al, 1995). However, the real impact of this approach remains to be confirmed in a randomized trial. At our institution, patients with SCLC are currently treated in the setting of such a trial, supported by the European Group for Blood and Marrow Transplantation (EBMT). This study compares a conventional regimen including lfosfamide-Carboplatinum-Etoposide (ICE) versus a sequential high-dose regimen with the same drugs followed by reinfusion of peripheral blood progenitor cells as described previously (Leyvraz et al, 1999).

*Received 9 April 2001*
*Revised 24 August 2001*
*Accepted 10 September 2001*

*Correspondence to:* L Perey

A potential limitation of this approach is the possibility to reinfuse tumour cells contaminating stem cell preparations. Tumour contamination of leukapheresis products is a commonly observed phenomenon in patients with solid tumours such as neuroblastoma or breast cancer that are characterized by extensive bone-marrow involvement (Moss et al, 1990; Ross et al, 1993). The clinical relevance of tumour cell reinfusion remains to be verified. Gribben et al have implicated reinfused tumour cells in post-transplant recurrence of bcl-2 positive lymphomas (Gribben et al, 1991). Using gene marking techniques, infused tumour cells were detected at sites of recurrence after autologous transplantation in patients with leukaemia (Brenner et al, 1993) and neuroblastoma (Rill et al, 1994).

In leukapheresis products from breast cancer patients, the preferred method of tracing contaminating tumour cells has been immunocytochemistry (ICC) (Ross et al, 1993; Passos-Coelho et al, 1996; Franklin et al, 1999). This immunolabelling assay relies on light microscopy of slides (cytospins). Other methods to replace or complement ICC have also been proposed, one of them being reverse transcriptase-polymerase chain reaction (RT-PCR) or quantitative RT-PCR, which can detect minute amounts of RNA. The target genes used to detect contamination of bone marrow or leukapheresis products with breast cancer cells have included epithelial markers such as cytokeratins (Passos-Coelho et al, 1996) (notably CK-19), carcinoembryonic antigen (CEA) or maspin (a serpin inhibitor) (Luppi et al, 1996). The data available on tumour cell contamination in leukapheresis products from patients with SCLC is sparse (Brugger et al, 1994). RT-PCR has never been used for this purpose. Therefore we performed the present study (i) to define appropriate markers for RT-PCR and (ii) to establish the usefulness of ICC as compared to RT-PCR in detecting tumour cell contamination of bone-marrow samples and leukapheresis products obtained from patients with small-cell lung cancer.

### MATERIALS AND METHODS

### Patients and controls

33 patients were included in the study. 16 patients participated in the sequential high-dose ICE program (Leyvraz et al, 1999) and had both baseline bone marrow and multiple leukapheresis assessments performed after mobilization chemotherapy, whereas the 17 remaining patients had only bone marrow assessments. The high-dose chemotherapy programme was approved by the Ethics Committee from the Faculté de Médecine, Lausanne University, Lausanne, Switzerland. In order to take part in this programme patients had to give informed consent. Patients not participating in the programme did not give informed consent before bone marrow sampling since this is considered as a routine procedure to determine disease extension at our institution. The median age of the patients was 60 years (range 40–67): They included 22 males (67%) and 11 females (33%). Staging was performed according to previously published recommendations (Stahel et al, 1989). 12 patients (36%) had limited disease and 21 (64%) had extensive disease. By definition, patients with limited disease showed no bone marrow contamination at cytology or histology. 8 patients with extensive disease had tumour cells in the bone marrow as demonstrated by routine light microscopy. In 6 patients, bone-marrow material was not available for ICC analysis or RT-PCR for technical reasons; however, histology and/or cytology confirmed massive involvement with tumour cells in all cases.

Haematopoietic progenitor cells were mobilized by 4-Epirubicin (Farmorubicin®, Pharmacia, Milan) administered intravenously at a dose of 75 mg $m^{-2}$ $day^{-1}$ for 2 days, followed by subcutaneous 5 µg $kg^{-1}$ G-CSF (Filgrastim, Roche, Basel, Switzerland) administered daily until the leukaphereses were completed. 30 samples collected from sequential leukaphereses (1–3 per patient) were analysed: 15 samples in 8 patients with limited disease and 15 samples in 8 patients with extensive disease. Bone marrow samples were collected from the posterior iliac crest with bilateral aspirations and unilateral biopsy. Conventional light microscopy was performed on aspiration material (cytology) and bone marrow biopsy (histology). Material from bilateral aspirations was pooled for ICC and RT-PCR analysis.

### Negative controls

10 blood samples from healthy volunteers and leukapheresis aliquots from 10 heathly allogeneic transplant donors were analysed. Mobilization in the transplant donors was performed by subcutaneous injections of G-CSF (filgrastim) 5 µg $kg^{-1}$. Bone marrow samples from patients with lymphomas, leukaemias and myeloma in remission were additionally used as controls for RT-PCR.

### Immunocytochemistry staining (ICC)

Mononuclear cells from bone marrow or leukapheresis products were isolated by density centrifugation through Ficoll/Hypaque (Pharmacia/Biotech, Uppsala, Sweden) at 460 ***g*** for 30 min. The cells in the interphase were washed twice in HBSS medium (Hank's balanced salts, Gibco BRL, Life Technologies, Basel, Switzerland). Samples containing $5 \times 10^5$ cells from bone marrow or leukapheresis products in HBSS medium were transferred onto glass slides using Hettich cytofunnels (area 240 $mm^2$) during centrifugation in a Universal Hettich 16 A centrifige (Hettich, Zurich, Switzerland). Cytospins were stained using a panel of antibodies in a 2-stage alkaline anti-alkaline phosphatase (APAAP) technique. The total number of cells examined per antibody was $1–2 \times 10^6$. The following monoclonal antibodies (MAb) were chosen for staining: MOC-1 (Bio-Science Products AG, Emmenbrücke, Switzerland), a MAb against neural-cell adhesion molecule (N-CAM); MOC31 (Bio-Science Products AG, Emmenbrücke, Switzerland), a MAb against EGP-40 (also known as EGP2 or EpCAM), a 38-kDa transmembrane glycoprotein expressed on the surface of most epithelial cells and the majority of carcinomas (Braun and Pantel, 1998); CAM5.2 (Becton Dickinson Biosciences, Allschwil, Switzerland), a MAb against cytokeratins 8 and 18 and finally MAb II-7 (Dako Diagnostics AG, Zug, Switzerland), which is specific for CEA. The following dilutions were chosen for staining; MOC1: 1/25; MOC31:1/60; CEA: 1/40, whereas CAM5.2 was used undiluted. All these antibodies showed low cross-reactivity with haematopoietic cells in bone marrow (< 1% stained cells). As negative controls, one cytospin slide was stained with mouse IgG1 (first antibody) and 2 cytospins with centrifuged KG-1A cells (a human acute myelogenous leukaemia cell line), were stained with MOC-1 and MOC-31. As positive controls we used SCLC cell line SW2 which expresses high levels of N-CAM, EGP-40, cytokeratins and CEA and yields bright staining with MAb MOC1, MOC31, CAM5.2 and MAb II-7. In cell seeding experiments with SW2 cells in mononuclear cells from bone marrow or leukapheresis products, MOC-1 staining detected one tumour cell per $10^5$ cells. The detection thresholds for MOC-31 were $1:10^5$ to $1:10^6$ cells.

### Markers for RT-PCR

SCLC exhibits many neuroendocrine properties on which we relied in our selection of RT-PCR markers. The following RNA transcripts were chosen based on protein expression by primary tumours and cells lines, as based on their use in ICC assays: N-CAM, synaptophysin, neuro-specific enolase and chromogranin (Blobel et al, 1985; Shy et al, 1990; Pasini et al, 1995). In addition to these markers, molecules selectively expressed by epithelium such as CK-18 and -19, CEA, EGP-40, MUC-1 (apomucin type 1) and ESM-1 (human endothelial cell-specific molecule) were also used (Hirsch et al, 1977; Broers et al, 1986, 1997; Beiske et al, 1992; Kim et al, 1992; Fields et al, 1996; Lassalle et al, 1996). The primers used for detecting these various transcripts are summarized in Table 1.

### Preparation of RNA and RT-PCR analysis

For RT-PCR assays, 2 ml of bone marrow or leukapheresis products were added to 7.5 ml TRI Reagent-BD (Molecular Research Center, Cincinnati, OH). The mixture was kept at -20˚C until RNA extraction. For some of the samples collected at the beginning of the study, only mononuclear cells (obtained after centrifugation on Ficoll/Hypaque gradient and kept at –80˚C) were available.

When the analysis was performed, total RNA was extracted from bone marrow or leukapheresis cells kept in the TRI Reagent-BD solution following the manufacturer's recommendations. Trizol (Life Technology, Gaithersburg, MD, USA) was also used to extract total RNA from mononuclear cells obtained after centrifugation on Ficoll/Hypaque gradient. After precipitation, the total RNA was

*© 2001 Cancer Research Campaign*

**Table 1** RT-PCR primers

| Gene | Primer sequence 5′–3′ | AT($MgCl_2$)[a] | PCR-cDNA |
|---|---|---|---|
| GAPDH | GGGAAGGTGAAGGTCGGAGTC<br>AGCAGAGGGGGCAGAGATGAT | 62 (1.5 mM) | 375 bp |
| p53 | TCTGGGCTTCTTGCATTCTGGGAA<br>TCTCGGAACATCTCGAAGCGCTCAC | 62 (1.5 mM) | 699 bp |
| CEA | AGC CCT GGT GTA GTT TCT TCA TT<br>AGA TGG GGT TTC ACG ATG TTG | 64 (1.8 mM) | 169 bp |
| CGA | TCCGCCGCTGTCCTGGCTCTTCT<br>TGGGGCTGGGCTTGGAAAGTGTGT | 60 (1.5 mM) | 142 bp |
| CK18 | ATG CCC GTC TTG CTG CTG AT<br>CTT GGC GAG GTC CTG AGA TTT G | 62 (1.5 mM) | 275 bp |
| CK19-outer | GCG GGC AAC GAG AAG CTA ACC<br>CTT CAG GCC TTC GAT CTG CAT | 64 (1.5 mM) | 495 bp |
| CK19-inner | CCCGCGACTACAGCCACTACTACAC<br>GCAGAGCCTGTTCCGTCTCAAA | 60 (1.5 mM) | 156 bp |
| CK20 | GCTCGGTGTGTCCTGCAAATTGATA<br>AGTGTTGCCCAGATGCTTGTGTAGG | 62 (1.5 mM) | 252 bp |
| Enolase | GAG CGG GCA GTG GAA GAA AAG G<br>GTC CGG CAA AGC GAG CTT CAT C | 64 (1.8 mM) | 292 bp |
| Ep-cam | GCG TTC GGG CTT CTG CTT GC<br>CCG CTC TCA TCG CAG TCA GGA | 62 (1.5 mM) | 287 bp |
| ESM1 | GGT GGA CTG CCC TCA ACA CT<br>AAG GTG CCG TAG GGA CAG TCT | 62 (1.8 mM) | 246 bp |
| MUC1 | ATC CCA GCA CCG ACT ACT ACC<br>AAG GAA ATG GCA CAT CAC TCA C | 60 (1.8 mM) | 264 bp |
| NCAM | GGA GGG GAA CCA GGT GAA CA<br>TGG TCG ATG GAT GGT GAA GAG | 58 (1.5 mM) | 270 bp |
| SYPH | TGC CAA CAA GAC CGA GAG TGA<br>CCA CAT GAA GGC GAA CAC AGC | 62 (1.5 mM) | 289 bp |

[a]AT: annealing temperature. $MgCl_2$ concentration in the PCR reaction is indicated in parentheses.
GAPDH = glyceraldehyde-3-phosphate dehydrogenase; CEA = carcinoembryonic antigen; CGA = chromogranin; CK = cytokeratin; EGP-40 = epidermal glycoprotein-40; ESM-1 = human endothelial cell-specific molecule; MUC-1 = apomucin type 1; NCAM = neural-cell adhesion molecule; SYPH = synaptophysin.

dissolved in 100 or 300 μl RNase-free water for bone marrow and leukapheresis products, respectively. The quantity of isolated RNA was determined by absorbance at 260 and 280 nm. RNA quality was assessed by analysis of about 0.6–1.0 mg of total RNA in a 1% agarose gel electrophoresis. When 28S and/or 18S ribosomal RNA (rRNA) bands appeared on the agarose gel, the rRNA was considered intact or partially degraded. When no 28S and 18S rRNA bands were present, the rRNA was considered totally degraded (Yan et al, 1998).

To eliminate contaminating DNA, 10 μg of RNA were exposed to RNAse-free DNase I (1 U $\mu g^{-1}$ RNA, Roche Diagnostics, Germany) at 37˚C for 30 minutes in 40 μl of a solution containing 40 mM Tris-HCL (pH7.9), 6 mM $MgCl_2$, 10 mM $CaCl_2$, 10 mM NaCl, 10 mM DTT and 4 U RNasine (Promega, Madison, WI, USA). Digestion was stopped by adding 5 μl of a stop-solution (50 mM EDTA and 1.5 M Na-acetate). The DNA-free RNA solution was re-extracted with phenol-chloroform and precipitated in ethanol in presence of 10 μg glycogen. cDNA was obtained from 5 μg of RNA DNA-free using Expand Reverse Transcriptase (RT) (Roche Diagnostics, Germany) and d(N)6 random primers as recommended by the manufacturer. A parallel RNA without Expand RT was run for each sample.

The resulting cDNA was subjected to PCR. Amplification reactions (20 μl) were performed in presence of 1/10 (2 μl) of the cDNA reaction, with an initial incubation step at 95˚C for 5 minutes. Cycling conditions consisted in denaturation at 94˚C for 30 seconds, annealing for 45 seconds, and extension at 72˚C for 45 seconds over a total of 40 cycles. The reaction was completed by another incubation step at 72˚C for 10 minutes. Annealing temperatures (AT) are indicated in Table 1. The reaction products were subjected to electrophoresis in 2% agarose gel and visualized by ethidium bromide staining. The RT-PCR procedure was performed at least twice for each sample. To ensure that the RT-PCR procedure was performed as required, an assay with primers specific for the glyceraldehyde-3-phosphate dehydrogenase (GAPDH) and p53 genes was run using the same cDNA aliquots.

### Statistical methods

Statistical comparisons of the different markers tested on the same samples were performed with the McNemar test for paired observations (Zar, 1974). Otherwise, comparisons between patients with limited versus extensive disease were performed with the Fisher test. For analyses of the tumour load in contaminated bone marrow and leukapheresis samples, the Kolmogorov–Smirnov non-parametric test for comparison of distributions was used (Conover, 1971).

### RESULTS

### Contamination of bone marrow samples as demonstrated by ICC staining

N-CAM-specific MAb MOC1, EGP-40-specific MAb MOC31 and cytokeratin-8/-18-specific MAb CAM5.2 were used for immunochemistry. Tumour cells were found in 13 of the 27 (48%)

© 2001 Cancer Research Campaign 

**Table 2** ICC staining results

| | N-CAM | EGP-40 | CK 8 + 18 |
|---|---|---|---|
| BM: | | | |
| LD ($N$ = 12) | 3 (25%) | 2 (17%) | 1 ( 8%) |
| ED ($n$ = 15) | 10 (67%) | 10 (67%) | 8 (53%) |
| Total ($n$ = 27) | 13 (48%) | 12 (44%) | 9 (33%) |
| LP: | | | |
| LD ($n$ = 15) | 4 (27%) | 0 ( 0%) | 0 ( 0%) |
| ED ($n$ = 14) | 6 (43%) | 0 ( 0%) | 0 ( 0%) |
| Total ($n$ = 29) | 10 (34%) | 0 ( 0%) | 0 ( 0%) |

ICC = Immunocytochemistry; BM = bone marrow; LP = leukaphereses; N-CAM = neural-cell adhesion molecule; EGP-40 = epithelial glycoprotein-40; CK 8+18 = cytokeratins 8+18; LD = Limited disease; ED = Extensive disease; $n$ = number of samples (several leukapheresis samples could have been collected from one patient).

analysed bone marrow samples. The results are broken down in Table 2. ICC staining revealed the presence of tumour cell in 3 of the 12 (25%) patients with limited disease. In the presence of extensive disease, by contrast, 10 of 15 (67%) patients showed bone marrow contamination. The difference of bone marrow contamination between limited and extensive disease was statistically significant ($P$ = 0.03). Immunostaining with mAb MOC1 against N-CAM was positive for all 13 positive bone marrow samples. MOC31 identified 12, CAM5.2 identified only 9 positive samples. The difference between contamination rates using the various antibodies was not statistically significant. An example of cells contaminating bone marrow is shown in Figure 2A and 2B.

CEA-specific mAb Il-7 was used on 10 bone-marrow samples. In 4 cases the result was negative although the presence of contamination was clearly established by cytology and/or histology. Staining patterns among positive cytospins were very heterogeneous. This low and variable expression by SCLC cells prompted us to abandon staining for CEA.

### Contamination of leukapheresis products as demonstrated by ICC staining

29 of the 30 leukapheresis samples were available for immunolabelling of cytospins. 10 of them (34%) were demonstrated to harbour contaminating tumour cells. Leukapheresis samples apparently only contained cells expressing N-CAM (Table 2). 4 of the 15 (27%) leukapheresis samples from patients with limited disease as compared to 6 of the 14 (43%) samples from patients with extensive disease were shown to be contaminated. This difference was not statistically significant ($P$ = 0.45). A contaminating cell in a leukapheresis product is shown in Figure 2C. Non-specific staining of a normal eosinophil is presented for comparison (Figure 2D). Morphological characteristics (cytoplasm and nucleus) are clearly different in the tumour and the haematopoietic cell.

6 patients with tumour cells in their leukapheresis products had sequential collections (on days 1 and 2): in 4 of them, contamination was observed in both leukapheresis harvests, while the remaining 2 patients showed tumour cells only in the first harvest. The possibility to enumerate stained cells enabled us to quantify tumour contamination on immunostained cytospins from bone marrow and leukapheresis products. In patients with extensive disease and contaminated bone marrow, a median of 15% of mononuclear cells (range 0.05–88%) could be identified as tumour cells. On the other hand, the number of tumour cells was much lower among the 3 patients with limited disease who had positive bone marrow, representing a median of 0.01% (range 0.001–0.2%). This difference approached statistical significance ($P$ = 0.075; Kolmogorov–Smirnov non-parametric test for comparison of distributions). Leukapheresis contamination was significantly less marked in patients with limited disease as compared to those with extensive disease: in the 6 extensive disease samples, the median contamination rate was 400 (range 10–700) tumour cells per $1 \times 10^6$ mononuclear cells (0.04%), whereas in the 4 limited disease samples a median of 10 (range 2–15) tumour cells per $1 \times 10^6$ mononuclear cells were identified ($P$ = 0.042; Kolmogorov–Smirnov non-parametric test).

We also compared bone marrow and leukapheresis contamination in the 16 patients who had leukaphereses. The presence of SCLC tumour cells in bone marrow as assessed by cytological or histological means or ICC staining was highly predictive of contaminating cells in leukapheresis products. Indeed, 5 out of the 6 patients (83%) with positive leukapheresis staining exhibited bone-marrow contamination. Of the 10 patients with negative leukaphereses, 6 (60%) showed no contamination of their bone marrow by standard examination and ICC staining. No contaminating tumour cells were observed in the leukapheresis products of patients who showed no bone marrow involvement.

### Appropriate markers for RT-PCR assessment of bone marrow and leukapheresis products

An appropriate marker for SCLC tumour cell detection in bone marrow and leukapheresis samples should return no positive signal (i) in the blood from healthy volunteers; (ii) in leukapheresis samples from normal subjects, i.e. healthy allogeneic transplant donors; (iii) in bone marrow from patients with haematopoietic malignancies such as lymphoma, myeloma or leukaemia. As apparent from Figure 1 and in Table 3, the criteria were met only by CK-19, CEA and chromogranin. Therefore we performed reconstitution experiments to determine the sensitivity of the RT-PCR for CK-19 and CEA, mixing 10-fold serial dilutions of different tumour cell lines (SW2, a small cell carcinoma cell line and SW480, a colorectal cancer cell line) with normal haematopoietic cells. With some of the cell lines tested, this yielded a sensitivity limit of approximately one tumour cell in $10^7$ normal cells for both markers. Some other cell lines, however, achieved only a sensitivity of $10^{-3}$, which indicates a wide range of CK-19 and CEA expression by epithelial cells.

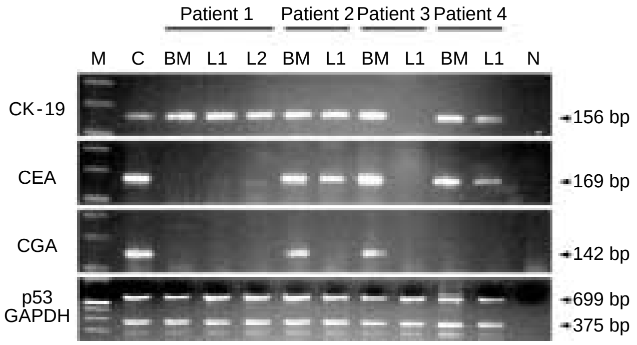

**Figure 1** RT-PCR signals in BM and LP. M = size marker (100 bp ladder); C = positive control cell line (SW2); BM = bone marrow; L1, L2 = sequential leukaphereses; N = negative control (RT-PCR performed in absence of RNA)

© 2001 Cancer Research Campaign

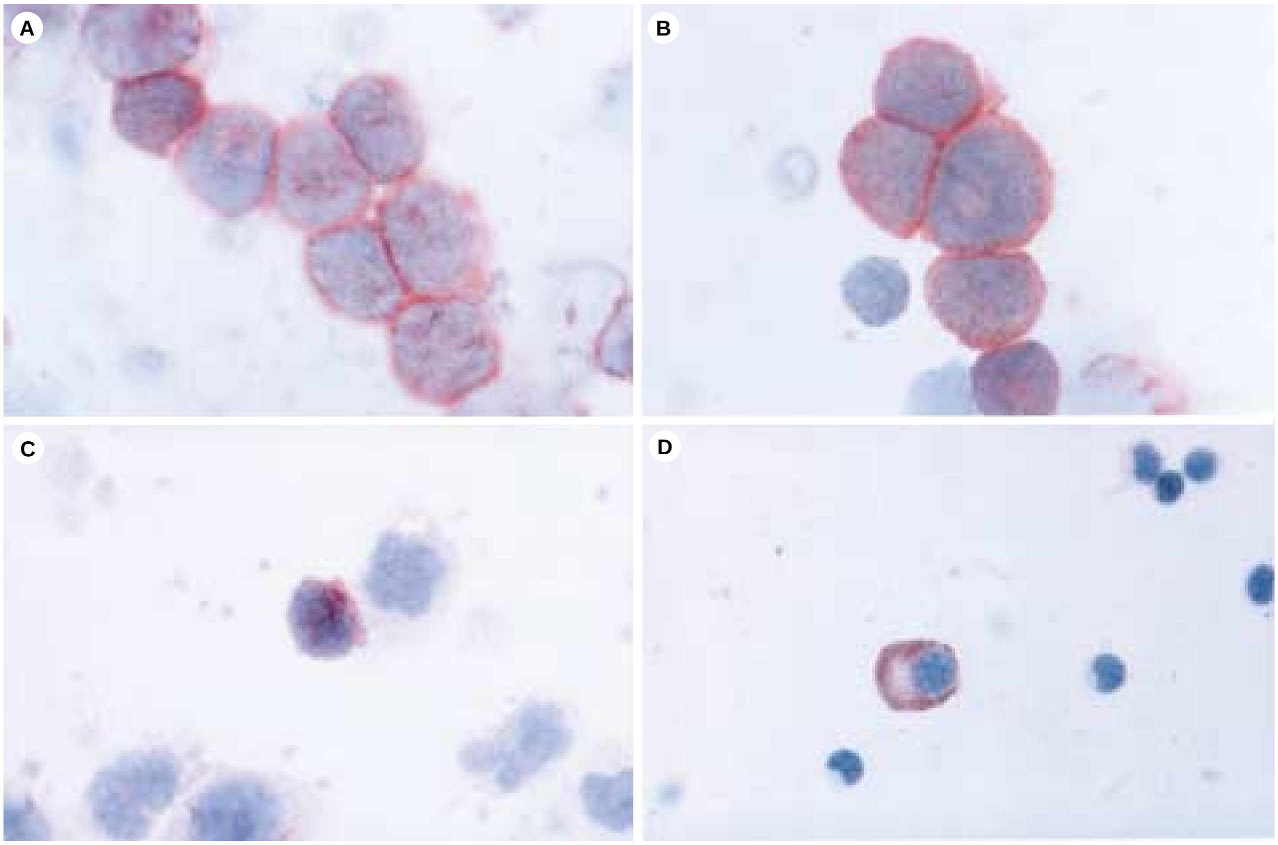

**Figure 2** Immunostaining of bone marrow and leukapheresis samples. (**A**) Cluster of contaminating cells in bone marrow (× 1000; MOC1 MAb staining against N-CAM, see Materials and Methods for dilution). (**B**) Cluster of contaminating cells in bone marrow (× 1000; MOC31 MAb staining against EGP-40). (**C**) Isolated contaminating tumour cell in a leukapheresis product (× 1000; MOC1 MAb staining against N-CAM). Note the high nuclear size/cytoplasm ratio. (**D**) Non-specific staining of a haematopoietic cell (eosinophil) in the bone marrow (× 500; MOC1 MAb staining against N-CAM). Note the morphology of the cell, the size of the stained granules and the nuclear size/cytoplasm ratio

**Table 3** RT-PCR markers

| | Blood[a] ($n$ = 10) | LP[b] ($n$ = 10) | BM[c] ($n$ = 12) |
|---|---|---|---|
| N-CAM, Enolase, CK-18, ESM-1, MUC-1 | Yes | Yes | Yes |
| EGP-40, Synaptophysin | No | Yes | Yes |
| CEA, CK-19, Chromogranin | No | No | No |

[a]Blood from healthy volunteers. [b]Leukaphereses from healthy donors. [c]Bone marrow from patients with haematological malignancies. BM = bone marrow; LP = leukaphereses; N-CAM = neural-cell adhesion molecule; EGP-40 = epithelial glycoprotein-40; CEA = carcinoembryonic antigen; CK 18/19 = cytokeratins 18 or 19; ESM-1 = human endothelial cell-specific molecule; MUC-1 = apomucin type 1.

**Table 4** RT-PCR results

| | CK-19 | CEA | CGA | RT-PCR (any positive signal) |
|---|---|---|---|---|
| BM: | | | | |
| LD ($n$ = 10) | 6 (60%) | 2 (20%) | 0 ( 0%) | 6 (60%) |
| ED ($n$ = 13) | 9 (69%) | 8 (62%) | 6 ( 50%) | 9 (69%) |
| Total ($n$ = 23) | 13 (48%) | 12 (44%) | 6 ( 26%) | 15 (65%) |
| LP: | | | | |
| LD ($n$ = 9) | 5 (56%) | 6 (67%) | 0 ( 0%) | 8 (89%) |
| ED ($n$ = 9) | 4 (44%) | 6 (67%) | 0 ( 0%) | 6 (67%) |
| Total ($n$ = 18) | 9 (50%) | 12 (67%) | 0 ( 0%) | 14 (78%) |

BM = bone marrow; LP = leukaphereses; CEA = carcinoembryonic antigen; CK 19 = cytokeratin 19; CGA = chromogranin; LD = Limited disease; ED = Extensive disease; $n$ = number of samples (several leukapheresis samples could have been collected from one patient).

### Contamination of bone marrow and leukapheresis products as demonstrated by RT-PCR

23 bone marrow samples were available for RT-PCR analysis. In the 10 patients with limited disease, positive signals were seen in 6 (60%) bone marrow samples with CK-19 primers, in 2 (20%) with CEA primers and in none with chromogranin (Table 4). Of the 13 patients with extensive disease, 9 (69%) showed positive bone marrow signals with CK-19, 8 (62%) with CEA and 6 out of 12 (50%) with chromogranin. Overall, positive signals were observed in 6 (60%) and 9 (69%) bone marrow samples from patients with limited and extensive disease respectively.

RT-PCR was also performed in 29 out of the 30 leukapheresis samples. In 11 samples, amplification of the control p53 and GAPDH mRNA transcripts did not result in clearly visible bands, thus reflecting poor RNA quality. We therefore considered that the results with CK19, CEA or chromogranin primers in those samples, even when positive, were not reliable and decided not to report them. All those samples had been collected at the beginning of the study. Mononuclear cells for RT-PCR analysis were obtained after centrifugation on Ficoll/Hypaque gradient. The duration of this procedure could have been deleterious to RNA

© 2001 Cancer Research Campaign 

transcripts. Solid data were obtained for the 18 remaining leukapheresis samples (Table 4). Of the 9 samples from patients with limited disease, positive signals were obtained in 5 (56%) cases after amplification with CD19 primers and in 6 (67%) cases after amplification with CEA primers, whereas chromogranin primers yielded no positive signals. Of the 9 samples from patients with extensive disease, 4 (44%) showed positive signals for CK-19 and 6 (67%) for CEA. Again, there were no positive signals after chromogranin amplification. Overall, positive signals were observed in 8 (89%) or 6 (67%) leukapheresis products from patients with limited or extensive disease, respectively.

The contamination patterns in bone marrow as compared to leukapheresis products could be assessed based on 7 patients. 5 of them had positive signals in leukapheresis products as determined by RT-PCR, all of them exhibiting visible bands in their bone-marrow samples. One patient was negative with regard to both bone marrow and leukapheresis, and one patient was characterized by negative stem-cell collections while the bone marrow sample expressed a positive signal. There were no cases of positive leukapheresis samples in the absence of bone-marrow contamination.

### ICC versus RT-PCR: comparison of results

The results obtained using RT-PCR in the 23 bone marrow samples were compared with those obtained by ICC in the same samples (Table 5). In 16 of them (70%) both results were in agreement, both for patients with limited (60%) and for patients with extensive disease (77%). Where results were not in agreement, it was in all cases but one the RT-PCR finding that was positive. Only one bone marrow sample that tested positive by ICC was not identified by RT-PCR, probably due to lack of CK-19, CEA and chromogranin expression by the tumour cells.

Likewise, we compared the results obtained by RT-PCR in the 18 leukapheresis samples to those observed with ICC assays (Table 5). Both results were in agreement in only 9 (50%) samples, again with no significant differences between patients with limited (44%) as compared to extensive (56%) disease. 7 out of these 9 (78%) stem cell collections with discordant results were positive by RT-PCR but negative by ICC, while the reverse was true in the remaining 2 patients (Table 5). Although the rate of agreement between the 2 methods appears to be lower in leukapheresis products (50%) than in bone marrow (70%), the number of samples was too small for this difference to reach statistical significance ($P = 0.2$).

Among the patients with limited disease, the rate of positive signals indicating contamination in bone marrow or leukapheresis products was much higher with RT-PCR (14/19, 74%) than with ICC (5/19, 26%). This difference was statistically significant ($P < 0.01$).

**Table 5** Comparison ICC staining and RT-PCR

| RT-PCR/ICC | BM LD ($n = 10$) | BM ED ($n = 13$) | LP LD ($n = 9$) | LP ED ($n = 9$) |
|---|---|---|---|---|
| Positive/positive | 2 | 7 | 3 | 4 |
| Negative/negative | 4 | 3 | 1 | 1 |
| Positive/negative | 4 | 2 | 5 | 2 |
| Negative/positive | 0 | 1 | 0 | 2 |
| Concordance | 6/10 (60%) | 10/13 (77%) | 4/9 (44%) | 5/9 (56%) |
| | Total: 16/23 (70%) | | Total: 9/18 (50%) | |

ICC = Immunocytochemistry staining; BM = bone marrow; LP = leukaphereses; LD = Limited disease; ED = Extensive disease.

### DISCUSSION

This is the first study addressing the use of RT-PCR assays for tumour cell detection in leukapheresis samples from patients with SCLC. The contamination rate of leukapheresis products as detected by RT-PCR based on CEA primers was 67% for patients with extensive disease. In patients with limited disease, positive signals were observed in 74% of cases. The rate of contamination identified by RT-PCR in leukapheresis products from patients with limited disease was significantly higher ($P < 0.01$) than the one observed by ICC staining. We also used RT-PCR assays for the first time to evaluate bone-marrow contamination. Positive signals based on chromogranin, CK19 or CEA primers were obtained in 50, 62% or 69%, respectively, of bone marrow samples from patients with extensive disease. In patients with limited disease, positive signals were observed in 60% of bone marrow samples based on CK-19 primers. This potential tumour contamination could explain the high incidence of relapse at distant sites observed in patients with limited disease. Our results also raise the question of assessing disease extension with more sensitive methods than light microscopy or ICC staining in patients with SCLC.

Contaminating tumour cells were clearly demonstrable by immunostaining in 34% of leukapheresis products and were more frequent (43 vs 27%) among patients with extensive than among those with limited disease. Also, the number of detected cells was significantly higher in patients with extensive disease. These results do not support previous findings in breast cancer patients where tumour cells have been observed in 10–20% of the leukapheresis products depending on the stage and extent of previous treatment (Ross et al, 1993; Passos-Coelho et al, 1996; Franklin et al, 1999). In a small study on SCLC, Brugger et al failed to detect tumour cells in peripheral blood of patients with limited disease (Brugger et al, 1994). By contrast, ICC staining with cytokeratins and an epithelium-specific cell surface antigen revealed 5 of 10 samples from extensive disease patients to be contaminated by tumour cells.

One of the advantages of immunostaining is that it allows quantifying the contaminating tumour cells. Thus we were able to demonstrate a significant difference of tumour load in the leukapheresis products of patients with limited as compared to extensive disease. Naturally the clinical relevance of these reinfused tumour cells remains to be verified in the same manner as it was for lymphoma, leukaemia or neuroblastoma (Gribben et al, 1991; Brenner et al, 1993; Rill et al, 1994). In the present study, among the patients participating in the high-dose chemotherapy programme, 10 had leukapheresis samples enabling a reliable RT-PCR analysis. 8 of them died of distant metastases. Numbers are too small to determine if leukapheresis contamination by ICC or RT-PCR was associated with a different time to progression or disease-free survival. 2 patients are still alive more than 3 years after diagnosis. one presented leukapheresis contamination by RT-PCR and the other not. Cells could not be detected by ICC in any leukapheresis of these 2 long survivors.

In the bone marrow, our results with antibodies specific for NCAM (48% of all patients, with 25% of patients with limited disease and 67% of patients with extensive disease) are consistent with the data in the literature (Hirsch et al, 1977; Stahel et al, 1985; Beiske et al, 1992; Pasini et al, 1995). Interestingly, contamination rates obtained with antibodies specifically directed against N-CAM,

*© 2001 Cancer Research Campaign*

EGP-40 or cytokeratins were similar. However, there was no need in the use of a cocktail of antibodies since sensitivity of tumour cell detection was not increased when pooling the results obtained with the various antibodies (data not shown). CEA, on the other hand, does not seem to be an appropriate marker for ICC staining in SCLC, as 4 out of 10 patients who clearly showed bone marrow contamination at routine light microscopy, tested negative with MAb II-7. Moreover, the results of ICC staining in the 6 remaining patients with CEA-positive tumour cells in their marrow varied widely between cells. Tumour cells in leukapheresis products were not detected by MAb MOC31 or CAM5.2 but were clearly identified by the N-CAM-specific MAb MOC1. Thus, it appears that antibodies that are useful for bone marrow might be inappropriate for the assessment of leukapheresis products.

RT-PCR has several potential advantages over ICC staining. Not only is the technique relatively easy to handle, but there are various tested primer sets available, cells can be analysed in great numbers, and the sensitivity is potentially higher than that of ICC assays (Schoenfeld et al, 1997; Ross, 1998; Zhong et al, 1999). On the downside, there also exist shortcomings of this method that prevent it from being universally accepted (Krismann et al, 1995; Bostick et al, 1998; Lambrechts et al, 1998). All PCR assays are extremely prone to contamination by all types of epithelial cells. The reported results are purely qualitative and there is no way to verify that the obtained signals are really due to tumour cell contamination. There is always a chance that pseudogenes or 'illegitimate transcripts' of some epithelial markers in haematopoietic cells (Datta et al, 1994; Krismann et al, 1995; Benhattar et al, 1998; Lambrechts et al, 1998) might interfere with the results.

It is not possible to choose a set of primers which would not amplify any of the potential pseudogenes. Indeed, these pseudogenes can vary from one population to another and, obviously, they are not all known. In order to overcome this limitation, several tools were used to avoid those pitfalls in the present study. To eliminate genomic DNA from the samples after RNA extraction, an extensive DNase 1 digestion was performed on the total extracted RNA. To exclude the possibility that signals were due to amplification of pseudogenes, a second RT-PCR with no reverse transcriptase added during the cDNA synthesis was always simultaneously run. Furthermore, we only used the most selective markers identified by extensive testing of negative controls – i.e. peripheral blood from healthy volunteers, as well as leukapheresis samples from normal allogeneic transplant donors and patients with haematological malignancies in remission. Our finding that CK-18, MUC-1 and EGP-40 were not suitable markers because of illegitimate transcription of targets in peripheral blood or bone marrow is consistent with previous reports (Helfrich et al, 1997; Lambrechts et al, 1998). Neuroendocrine markers such as N-CAM, enolase and synaptophysin had to be further excluded due to 'illegitimate transcripts' within haematopoietic cells.

Unlike previous investigators (Krismann et al, 1995; Zippelius et al, 1997), we observed no false-positive results with CEA or CK-19 primer amplification in our various negative controls. We suspect that the results of those studies may have been distorted by the presence of pseudogenes. To verify this notion, we performed a nested PCR after DNase digestion, with the outer CK-19 primers followed by an amplification with the inner CK-19 primers, which confirmed the absence of signals even when PCR cycles were significantly increased (data not shown). It therefore appears that our results obtained in bone-marrow and leukapheresis samples indeed reflect real contamination by tumour cells.

The results obtained with either ICC staining or RT-PCR were consistent in 50% of leukapheresis samples. Where they conflicted, it was in 78% of cases the RT-PCR that was positive and the ICC staining that was negative. This difference could be due to the sensitivity of both assays, which some authors have claimed is higher with RT-PCR (Schoenfeld et al, 1997; Zhong et al, 1999). In our study, there was no way for us to compare the sensitivities involved because the number of mononuclear cells used with both methods was not standardized. Also, it was not possible to use the same markers in all tests. Bone-marrow and leukapheresis products definitely differ in terms of cell environment and cell stimulation. Indeed we observed that some markers (like EGP-40 and cytokeratins with ICC staining and chromogranin with RT-PCR) were no longer detectable in leukapheresis products after mobilization chemotherapy and G-CSF. A similar phenomenon was described by Krüger et al, who found bone-marrow samples from normal donors to be negative for CK19, whereas cultured bone-marrow cells would express this marker (Krüger et al, 1998). These results suggest that it is possible under certain conditions to induce transcription of 'epithelium-specific markers' in haematopoietic tissue. Furthermore they indicate that a good tumour cell marker in bone marrow is not automatically a good choice when it comes to analysing leukapheresis products.

ICC assays are still considered the 'gold standard' in detecting tumour cell contamination, as the morphology of stained cells can be evaluated under the microscope and the degree of contamination quantified. By comparison, RT-PCR assays are potentially more effective in assessing the real contamination of leukapheresis samples in patients with SCLC. In bone-marrow samples, the results obtained with ICC and RT-PCR are relatively consistent (general agreement 70%) provided that appropriate precautions are taken to avoid false-positive and false-negative results. In leukapheresis products, the rate of agreement between ICC and RT-PCR is 50%, reflecting a greater number of positive findings obtained with RT-PCR, which confirms that RT-PCR is preferable for leukapheresis products. In our hands, CEA has proved to be the best marker in this context. The clinical implications of this phenomenon will be studied further in a multicentre randomized phase III trial supported by the European Bone Marrow Transplant (EBMT) group, which will compare a standard chemotherapeutic approach with a sequential high-dose regimen supplemented with autologous haematopoietic progenitors.

### ACKNOWLEDGEMENTS

The authors wish to thank Gabrielle Gallagher for her excellent technical assistance. They are indebted to S Pampallona (ForMed, Evolène, Suisse) for conducting the statistical analysis. This study was supported by grants from the *R*echerche Suisse contre le Cancer (KFS 0195-9-1995 and KFS 561-9-1997) and by a grant from La Ligue Vaudoise contre le cancer.

### REFERENCES

Arriagada R, Le Chevalier T, Pignon JP, Riviere A, Monnet I, Chomy P, Tuchais C, Tarayre M and Ruffie P (1993) Initial chemotherapeutic doses and survival in patients with limited small-cell lung cancer. *N Engl J Med* **329**: 1848–1852

Beiske K, Myklebust AT, Aamdal S, Langholm R, Jakobsen E and Fodstad O (1992) Detection of bone marrow metastases in small cell lung cancer patients. Comparison of immunologic and morphologic methods. *Am J Pathol* **141**: 531–538

© 2001 Cancer Research Campaign 

Benhattar J, Perey L, Peters R and Leyvraz S (1998) Reverse transcriptase-polymerase chain reaction and islet cell carcinoma: not antagonist, but complementary tools (letter; comment). *J Clin Oncol* **16**: 806–807

Blobel GA, Gould VE, Moll R, Lee I, Huszar M, Geiger B and Franke WW (1985) Coexpression of neuroendocrine markers and epithelial cytoskeletal proteins in bronchopulmonary neuroendocrine neoplasms. *Lab Invest* **52**: 39–51

Bostick PJ, Chatterjee S, Chi DD, Huynh KT, Giuliano AE, Cote R and Hoon DS (1998) Limitations of specific reverse-transcriptase polymerase chain reaction markers in the detection of metastases in the lymph nodes and blood of breast cancer patients. *J Clin Oncol* **16**: 2632–260

Braun S and Pantel K (1998) Prognostic significance of micrometastatic bone marrow involvement. *Breast Cancer Res Treat* **52**: 201–216

Brenner MK, Rill DR, Moen RC, Krance RA, Mirro J Jr., Anderson WF and Ihle JN (1993) Gene-marking to trace origin of relapse after autologous bone-marrow transplantation. *Lancet* **341**: 85–86

Broers JL, Carney DN, Klein Rot M, Schaart G, Lane EB, Vooijs GP and Ramaekers FC (1986) Intermediate filament proteins in classic and variant types of small cell lung carcinoma cell lines: a biochemical and immunochemical analysis using a panel of monoclonal and polyclonal antibodies. *J Cell Sci* **83**: 37–60

Broers JL, Rot MK, Oostendorp T, Huysmans A, Wagenaar SS, Wiersma-van Tilburg AJ, Vooijs GP and Ramaekers FC (1987) Immunocytochemical detection of human lung cancer heterogeneity using antibodies to epithelial, neuronal, and neuroendocrine antigens. *Cancer Res* **47**: 3225–3234

Brugger W, Bross KJ, Glatt M, Weber F, Mertelsmann R and Kanz L (1994) Mobilization of tumor cells and hematopoietic progenitor cells into peripheral blood of patients with solid tumors (see comments). *Blood* **83**: 636–640

Conover WJ (1971) In *Practical nonparametric statistics*. John Wiley and Sons: New York

Datta YH, Adams PT, Drobyski WR, Ethier SP, Terry VH and Roth MS (1994) Sensitive detection of occult breast cancer by the reverse-transcriptase polymerase chain reaction. *J Clin Oncol* **12**: 475–482

Ettinger DS, Karp JE, Abeloff MD, Burke PJ and Braine HG (1978) Intermittent high-dose cyclophosphamide chemotherapy for small cell carcinoma of the lung. *Cancer Treat Rep* **62**: 413–424

Farha P, Spitzer G, Valdivieso M, Dicke KA, Zander A, Dhingra HM, Minnhaar G, Vellekoop L, Verma DS, Umsawasdi T et al (1983) High-dose chemotherapy and autologous bone marrow transplantation for the treatment of small cell lung carcinoma. *Cancer* **52**: 1351–1355

Fields KK, Elfenbein GJ, Trudeau WL, Perkins JB, Janssen WE and Moscinski LC (1996) Clinical significance of bone marrow metastases as detected using the polymerase chain reaction in patients with breast cancer undergoing high-dose chemotherapy and autologous bone marrow transplantation. *J Clin Oncol* **14**: 1868–1876

Franklin WA, Glaspy J, Pflaumer SM, Jones RB, Hami L, Martinez C, Murphy JR and Shpall EJ (1999) Incidence of tumor-cell contamination in leukapheresis products of breast cancer patients mobilized with stem cell factor and granulocyte colony-stimulating factor (G-CSF) or with G-CSF alone. *Blood* **94**: 340–347

Gribben JG, Freedman AS, Neuberg D, Roy DC, Blake KW, Woo SD, Grossbard ML, Rabinowe SN, Coral F, Freeman GJ et al (1991) Immunologic purging of marrow assessed by PCR before autologous bone marrow transplantation for B-cell lymphoma (see comments). *N Engl J Med* **325**: 1525–1533

Helfrich W, ten Poele R, Meersma GJ, Mulder NH, de Vries EG, de Leij L and Smit EF (1997) A quantitative reverse transcriptase polymerase chain reaction-based assay to detect carcinoma cells in peripheral blood. *Br J Cancer* **76**: 29–35

Hirsch F, Hansen HH, Dombernowsky P and Hainau B (1977) Bone-marrow examination in the staging of small-cell anaplastic carcinoma of the lung with special reference to subtyping. An evaluation of 203 consecutive patients. *Cancer* **39**: 2563–2567

Kim J, Kaye FJ, Henslee JG, Shively JE, Park JG, Lai SL, Linnoila RI, Mulshine JL and Gazdar AF (1992) Expression of carcinoembryonic antigen and related genes in lung and gastrointestinal cancers. *Int J Cancer* **52**: 718–725

Krismann M, Todt B, Schroder J, Gareis D, Muller KM, Seeber S and Schutte J (1995) Low specificity of cytokeratin 19 reverse transcriptase-polymerase chain reaction analyses for detection of hematogenous lung cancer dissemination. *J Clin Oncol* **13**: 2769–2775

Krüger WH, Jung R, Hennings S, Kröger N and Zander A (1998) Cytokine-influence on specificity of cytokeratin-19 RT-PCR for cancer cell detection. *Bone Marrow Transplant* **21**: S185

Lambrechts AC, van't Veer LJ and Rodenhuis S (1998) The detection of minimal numbers of contaminating epithelial tumor cells in blood or bone marrow: use, limitations and future of RNA-based methods. *Ann Oncol* **9**: 1269–1276

Lassalle P, Molet S, Janin A, Heyden JV, Tavernier J, Fiers W, Devos R and Tonnel AB (1996) ESM-1 is a novel human endothelial cell-specific molecule expressed in lung and regulated by cytokines. *J Biol Chem* **271**: 20458–20464

Leyvraz S, Perey L, Rosti G, Lange A, Pampallona S, Peters R, Humblet Y, Bosquee L, Pasini F and Marangolo M (1999) Multiple courses of high-dose ifosfamide, carboplatin, and etoposide with peripheral-blood progenitor cells and filgrastim for small-cell lung cancer: A feasibility study by the European Group for Blood and Marrow Transplantation. *J Clin Oncol* **17**: 3531–3539

Luppi M, Morselli M, Bandieri E, Federico M, Marasca R, Barozzi P, Ferrari MG, Savarino M, Frassoldati A and Torelli G (1996) Sensitive detection of circulating breast cancer cells by reverse-transcriptase polymerase chain reaction of maspin gene. *Ann Oncol* **7**: 619–624

Moss TJ, Sanders DG, Lasky LC and Bostrom B (1990) Contamination of peripheral blood stem cell harvests by circulating neuroblastoma cells. *Blood* **76**: 1879–1883

Muggia FM, Krezoski SK and Hansen HH (1974) Cell kinetic studies in patients with small cell carcinoma of the lung. *Cancer* **34**: 1683–1690

Pasini F, Pelosi G, Mostacci R, Santo A, Masotti A, Spagnolli P, Recaldin E and Cetto GL (1995) Detection at diagnosis of tumor cells in bone marrow aspirates of patients with small-cell lung cancer (SCLC) and clinical correlations. *Ann Oncol* **6**: 86–88

Passos-Coelho JL, Ross AA, Kahn DJ, Moss TJ, Davis JM, Huelskamp AM, Noga SJ, Davidson NE and Kennedy MJ (1996) Similar breast cancer cell contamination of single-day peripheral-blood progenitor-cell collections obtained after priming with hematopoietic growth factor alone or after cyclophosphamide followed by growth factor. *J Clin Oncol* **14**: 2569–2575

Pettengell R, Woll PJ, Thatcher N, Dexter TM and Testa NG (1995) Multicyclic, dose-intensive chemotherapy supported by sequential reinfusion of hematopoietic progenitors in whole blood. *J Clin Oncol* **13**: 148–156

Rill DR, Santana VM, Roberts WM, Nilson T, Bowman LC, Krance RA, Heslop HE, Moen RC, Ihle JN and Brenner MK (1994) Direct demonstration that autologous bone marrow transplantation for solid tumors can return a multiplicity of tumorigenic cells. *Blood* **84**: 380–383

Ross AA (1998) Minimal residual disease in solid tumor malignancies: a review. *J Hematother* **7**: 9–18

Ross AA, Cooper BW, Lazarus HM, Mackay W, Moss TJ, Ciobanu N, Tallman MS, Kennedy MJ, Davidson NE, Sweet D et al (1993) Detection and viability of tumor cells in peripheral blood stem cell collections from breast cancer patients using immunocytochemical and clonogenic assay techniques (see comments). *Blood* **82**: 2605–2610

Schoenfeld A, Kruger KH, Gomm J, Sinnett HD, Gazet JC, Sacks N, Bender HG, Luqmani Y and Coombes RC (1997) The detection of micrometastases in the peripheral blood and bone marrow of patients with breast cancer using immunohistochemistry and reverse transcriptase polymerase chain reaction for keratin 19. *Eur J Cancer* **33**: 854–861

Shy SW, Lee WH, Chou MC, Lai YS and Tu YC (1990) Small cell lung carcinoma: clinicopathological, immunohistochemical, and ultrastructural study. *J Surg Oncol* **45**: 146–161

Souhami RL, Finn G, Gregory WM, Birkhead BG, Buckman R, Edwards D, Goldstone AH, Harper PG, Spiro SG, Tobias JS et al (1985) High-dose cyclophosphamide in small-cell carcinoma of the lung. *J Clin Oncol* **3**: 958–963

Stahel RA, Mabry M, Skarin AT, Speak J and Bernal SD (1985) Detection of bone marrow metastasis in small-cell lung cancer by monoclonal antibody. *J Clin Oncol* **3**: 455–461

Stahel RA, Ginsberg R, Havermann K, Hirsch FR, Ihde DC, Jassem J, Karrer K, Maurer LM, Osterlind K and P VH (1989) Staging and prognostic factors in small cell lung: a consensus report. *Lung Cancer* **5**: 119–126

Steward WP, von Pawel J, Gatzemeier U, Woll P, Thatcher N, Koschel G, Clancy L, Verweij J, de Wit R, Pfeifer W, Fennelly J, von Eiff M and Frisch J (1998) Effects of granulocyte-macrophage colony-stimulating factor and dose intensification of V-ICE chemotherapy in small-cell lung cancer: a prospective randomized study of 300 patients. *J Clin Oncol* **16**: 642–650

Thatcher N, Girling DJ, Hopwood P, Sambrook RJ, Qian W and Stephens RJ (2000) Improving survival without reducing quality of life in small-cell lung cancer patients by increasing the dose-intensity of chemotherapy with granulocyte colony-stimulating factor support: results of a British Medical Research Council Multicenter Randomized Trial. Medical Research Council Lung Cancer Working Party. *J Clin Oncol* **18**: 395–404

Weiss RB (1978) Small-cell carcinoma of the lung: therapeutic management. *Ann Intern Med* **88**: 522–531

Weiss W, Boucot KR and Cooper DA (1970) The histopathology of bronchogenic carcinoma and its relation to growth rate, metastasis, and prognosis. *Cancer* **26**: 965–970

Woll PJ, Hodgetts J, Lomax L, Bildet F, Cour-Chabernaud V and Thatcher N (1995) Can cytotoxic dose-intensity by increased by using granulocyte colony-stimulating factor? A randomized controlled trial of lenograstim in small-cell lung cancer. *J Clin Oncol* **13**: 652–659

*© 2001 Cancer Research Campaign*

Yan P, Bosman FT and Benhattar J (1998) Tissue quality is an important determinant of telomerase activity as measured by TRAP assay. *Biotechniques* **25**: 660–662

Zar JH (1974) In *Biostatistical Analysis*, Prentice-Hall (ed) pp. 127: Englewood Cliffs, NJ

Zhong XY, Kaul S, Diel I, Eichler A and Bastert G (1999) Analysis of sensitivity and specificity of cytokeratin 19 reverse transcriptase/polymerase chain reaction for detection of occult breast cancer in bone marrow and leukapheresis products. *J Cancer Res Clin Oncol* **125**: 286–291

Zippelius A, Kufer P, Honold G, Kollermann MW, Oberneder R, Schlimok G, Riethmuller G and Pantel K (1997) Limitations of reverse-transcriptase polymerase chain reaction analyses for detection of micrometastatic epithelial cancer cells in bone marrow [see comments]. *J Clin Oncol* **15**: 2701–2708

© 2001 Cancer Research Campaign
